# Monoacylglycerol Lipase Knockdown Inhibits Cell Proliferation and Metastasis in Lung Adenocarcinoma

**DOI:** 10.3389/fonc.2020.559568

**Published:** 2020-12-09

**Authors:** Hao Zhang, Wei Guo, Fan Zhang, Renda Li, Yang Zhou, Fei Shao, Xiaoli Feng, Fengwei Tan, Jie Wang, Shugeng Gao, Yibo Gao, Jie He

**Affiliations:** ^1^ Department of Thoracic Surgery, National Cancer Center/National Clinical Research Center for Cancer/Cancer Hospital, Chinese Academy of Medical Sciences and Peking Union Medical College, Beijing, China; ^2^ The Affiliated Hospital of Qingdao University and Qingdao Cancer Institute, Qingdao, China; ^3^ Department of Pathology, National Cancer Center/National Clinical Research Center for Cancer/Cancer Hospital, Chinese Academy of Medical Sciences and Peking Union Medical College, Beijing, China; ^4^ State Key Laboratory of Molecular Oncology, Department of Medical Oncology, National Cancer Center/National Clinical Research Center for Cancer/Cancer Hospital, Chinese Academy of Medical Sciences and Peking Union Medical College, Beijing, China

**Keywords:** monoacylglycerol lipase, matrix metalloproteinase 14, proliferation, metastasis, lung adenocarcinoma, prognosis

## Abstract

Abnormal metabolism is one of the hallmarks of cancer cells. Monoacylglycerol lipase (MGLL), a key enzyme in lipid metabolism, has emerged as an important regulator of tumor progression. In this study, we aimed to characterize the role of MGLL in the development of lung adenocarcinoma (LUAD). To this end, we used tissue microarrays to evaluate the expression of MGLL in LUAD tissue and assessed whether the levels of this protein are correlated with clinicopathological characteristics of LUAD. We found that the expression of MGLL is higher in LUAD samples than that in adjacent non-tumor tissues. In addition, elevated MGLL expression was found to be associated with advanced tumor progression and poor prognosis in LUAD patients. Functional studies further demonstrated that stable short hairpin RNA (shRNA)-mediated knockdown of MGLL inhibits tumor proliferation and metastasis, both *in vitro* and *in vivo*, and mechanistically, our data indicate that MGLL regulates Cyclin D1 and Cyclin B1 in LUAD cells. Moreover, we found that knockdown of MGLL suppresses the expression of matrix metalloproteinase 14 (MMP14) in A549 and H322 cells, and in clinical samples, expression of MMP14 is significantly correlated with MGLL expression. Taken together, our results indicate that MGLL plays an oncogenic role in LUAD progression and metastasis and may serve as a potential biomarker for disease prognosis and as a target for the development of personalized therapies.

## Introduction

Non-small cell lung cancer is one of the leading causes of cancer-related deaths worldwide, with lung adenocarcinoma (LUAD) representing the major subtype of this disease (1, 2). Although significant progress has been made in the treatment of LUAD, it is still associated with a high mortality rate (3). In particular, a number of new target drugs have been discovered and applied for patients with lung cancer in recent years. However, the sensitivity to these drugs varies extensively among patients (4).

Emerging evidences have revealed that dysregulated metabolic pathways involved in cancer development (5, 6), and metabolic reprogramming can regulate cancer cell stemness, proliferation, metastasis, and drug resistance (7, 8). Notably, tumor progression and metastasis are recognized as main causes of death in patients with lung cancer (9, 10). Recently, a number of studies have further demonstrated that aberrantly activated lipid metabolic pathways can promote cancer cell proliferation and metastasis (11–13), and intriguingly, disordered lipid metabolism has been detected in lung cancer (14). These observations therefore suggest that the development of drugs targeting lipid metabolic pathways may provide a new therapeutic strategy for LUAD.

Monoacylglycerol lipase (MGLL) is an important enzyme in the process of lipolysis, which functions to hydrolyze monoacylglycerol (MAG), producing glycerol and free fatty acids (FFA) (15, 16). In this way, MGLL can regulate a number of cellular processes, as both MAGs and FFA can act as signaling lipids or precursors (15). Critically, MGLL has also been found to contribute to tumorigenesis and cancer metastasis. Studies have revealed that MGLL is highly expressed in a number of aggressive human cancer cells and primary tumors, and overexpression of MGLL promotes tumor migration, invasion, and proliferation (16–18). However, the role of MGLL in lung cancer has not been elucidated.

Here, to address this question, we measured and compared MGLL expression in LUAD samples and adjacent non-tumor tissues. We then established cell lines with stable depletion of MGLL to assess the effect of MGLL on malignant phenotypes of LUAD cells both *in vitro* and *in vivo*. Lastly, we explored the mechanisms by which MGLL can modulate cell proliferation and metastasis in LUAD.

## Materials and Methods

### Cell Lines and Cell Culture

H322 and A549 LUAD cell lines were cultivated in Dulbecco’s Modified Eagle Medium (DMEM) and Roswell Park Memorial Institute (RPMI) 1640 medium (Corning, Corning, NY, USA), respectively, supplemented with 10% fetal bovine serum (Corning, Mediatech Inc., Manassas, VA, USA). Cell lines were cultured at 37°C with 5% CO_2_. Moreover, all the cell lines used in the study were regularly authenticated by short tandem repeat detection and tested for the presence of mycoplasma.

### Patient Samples

Formalin-fixed paraffin-embedded cancer tissues from 156 patients diagnosed with LUAD between 2006 and 2017, as well as 76 adjacent non-tumor tissues, were obtained from the Cancer Hospital, Chinese Academy of Medical Sciences. Information including age, gender, and tumor node metastasis (TNM) stage was collected from medical records. Informed consent was obtained from all patients and the study protocol was approved by the Research Ethics Committee of the Cancer Hospital, Chinese Academy of Medical Sciences.

### Immunohistochemical Staining

Sections of paraffin-embedded LUAD and adjacent non-tumor tissues were prepared for immunohistochemical staining. Briefly, antigen retrieval was performed by microwaving the sections in Antigen Retrieval Buffer with pH 6.0. Sections were incubated with MGLL (1:200, Proteintech) or MMP14 (1:200, Abclonal) antibodies overnight at 4°C and then incubated with appropriate secondary antibodies. We quantitatively scored the tissues according to the percentage of positive staining cells and the staining intensity. Briefly, the staining intensity was graded as 0 (negative), 1 (low), 2 (moderate) or 3 (high), and the proportion of staining was evaluated as 0 (negative), 1 (< 1%), 2 (1–10%), 3 (11–30%), 4 (31–70%), or (71–100%). A pathologist and two experienced researchers independently scored the slides.

### Establishment of Stable Knockdown Cell Lines

Two short-hairpin RNAs, 5’- GGATGGTAGTGTCTGACTTCC -3’ and 5’- CAACTCCGTCTTCCATGAAAT-3’, were synthesized for MGLL suppression. They were independently inserted into the pLKO.1-puro lentiviral shRNA vector (Generay Biotech Co., Shanghai, China) to produce lentivirus in HEK293T cells. Cells were selected using medium containing 2 μg/ml puromycin (Sigma-Aldrich, USA) for 7 days.

### Quantitative Reverse Transcription PCR (qRT-PCR)

qRT-PCR was performed as previously described (19). The primers used for MGLL: forward, 5’- CACAGTGGCCGCTATGAAGA-3’; reverse, 5’- CCACATGCTGCAACACATCC-3’.

### Western Blot

Western blots were performed as previously described (20). The following primary antibodies were used: MGLL (1:200, Abcam, Cambridge, UK), Tubulin (1:5,000, Sigma-Aldrich), CCNB1 (1:1,000, Cell Signaling Technology, Danvers, MA, USA), CCND1 (1:1,000, Cell Signaling Technology), BCL-2 (1:1,000, Cell Signaling Technology), BAX (1:1000, Cell Signaling Technology), E-cadherin (1:1,000, Cell Signaling Technology), N-cadherin (1:1,000, Cell Signaling Technology), and MMP14 (1:500, ABclonal).

### Proliferation and Colony Formation Assay

Cell viability was assessed by the cell counting kit-8 (CCK-8) assay (Dojindo, Kumamoto, Japan). Optical density was measured at 450 nm, and the cells were monitored continuously for 3 days. For colony formation assays, A549 and H322 cells were seeded in 6-well plates at 600 cells/well, and these were allowed to grow for 1 and 2 weeks, respectively. The colonies were then fixed and stained with 1% crystal violet.

### Migration and Invasion Assay

For migration and invasion assays, A549 cells (5 × 10^4^) or H322 cells (1 × 10^5^) in serum-free RPMI 1640 or DMEM medium, respectively, were plated into the upper chamber of 24-well transwell inserts (Corning, 8.0-μm pores) that were either uncoated or coated with Matrigel (BD Biosciences, Franklin Lakes, NJ, USA). Experiments were performed as previously described (21).

### Cell Cycle and Cell Apoptosis Analysis

For cell cycle analysis, cells were harvested and fixed with 70% alcohol at 4°C overnight, digested in RNase at 37°C for 30 min, stained with propidium iodide (PI) for 30 min and analyzed with a BD flow cytometer (Becton Dickinson FACSCanto II). For cell apoptosis analysis, cells were digested, washed and then resuspended with binding buffer. Then, FITC annexin V and PI were added to the cell suspension and incubated in the dark for 10 min. Apoptosis was detected with a BD FACSCanto II flow cytometer.

### RNA Sequencing (RNA-seq)

RNA-seq was used to measure mRNA expression profiles after knockdown of MGLL in A549 cells. For these assays, total RNA extraction, cDNA library preparation, and RNA sequencing were performed at Novogene (Beijing, China). Differentially expressed genes (DEGs) were identified with Cuffdiff, using a cutoff of |fold change| ≥2.0 as our threshold for identifying upregulated and downregulated mRNAs (Supplementary Data 1).

### Animal Experiments

BALB/c-nu mice (female, 4–5 weeks old) were used for our xenograft model. Briefly, A549 cells transfected with MGLL-shRNA or the vector control were injected into the right dorsal flanks of BALB/c-nu mice (1.5 × 10^6^ cells per animal, 6 mice per group). Tumor size was measured twice a week. All mice were sacrificed after 4 weeks, and the tumors were excised and weighed. Non-obese diabetic (NOD)-SCID mice (female, 4–5 weeks old) were used for our lung metastasis model. For these experiments, A549 cells (1 × 10^6^ cells per animal, 6 mice per group) transfected with MGLL-shRNA or the vector control were administered to NOD-SCID mice *via* tail vein injection. After 8 weeks, the mice were sacrificed, lungs were excised, and the number of lung metastatic nodules was evaluated by gross and microscopic examination.

### Statistics

Data analysis was performed using GraphPad Prism 7 (GraphPad Software, Inc., San Diego, CA, USA). The chi-square test was used to identify associations between clinicopathological characteristics and MGLL expression. Survival analysis was performed with the Kaplan-Meier method and the log-rank test. Data were compared between groups using two-tailed Student’s *t*-tests, and the results were expressed as the mean ± standard deviation (SD). *P* <0.05 was considered statistically significant.

## Results

### MGLL Is Significantly Overexpressed in LUAD Tissue and Increased MGLL Levels Are Correlated With Poor Prognosis

**Figure 1:**
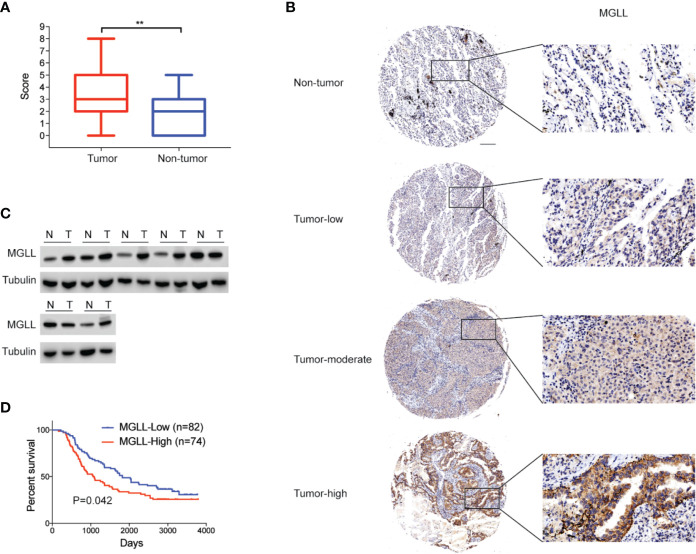
MGLL is overexpressed in LUAD tissue, and its expression levels are correlated with poor prognosis. **(A)** Immunohistochemical (IHC) staining reveals that MGLL is overexpressed in LUAD relative to adjacent non-tumor tissues (**P < 0.01). **(B)** Representative images from IHC staining of MGLL in LUAD and non-tumor tissues; scale bar = 50 µm. **(C)** Western blot analysis of MGLL expression in randomly selected LUAD samples and adjacent non-tumor tissues. **(D)** Kaplan-Meier analysis indicates that high MGLL expression is predictive of poor overall survival.

We first used IHC staining to detect MGLL expression in 156 LUAD samples and 76 adjacent non-tumor tissues and found that MGLL expression is significantly overexpressed in cancer tissues relative to non-tumor tissues (**Figures 1A, B**). Our data further suggest that MGLL is mainly localized to the cytoplasm in both LUAD and non-tumor tissues (**Figure 1B**). We then conducted western blot analysis to measure MGLL levels in seven pairs of randomly selected LUAD and non-tumor samples, and our data confirm that most tumor tissues display elevated MGLL expression, as compared to adjacent non-tumor tissues (**Figure 1C**).

We further investigated the associations between MGLL levels and the clinicopathological characteristics of LUAD patients (**Table 1**). The chi-square test showed that the expression level of MGLL is significantly correlated with TNM stage (P = 0.041) and histology grade (P = 0.037). Furthermore, we examined the relationship between MGLL expression level and patient survival. MGLL expression level was significantly correlated with patient survival, and patients high MGLL expression had worse outcomes according to both Kaplan-Meier analysis (P = 0.042, log-rank test; **Figure 1D**) and univariate Cox regression analysis (**Supplementary Table 1**). However, MGLL expression was not independently associated with overall survival by multivariate Cox regression analysis in this cohort after adjustment of age, histology grade and TNM stage (**Supplementary Table 1**).

**Table 1:** Correlations between MGLL expression and clinicopathological characteristics of LUAD patients.

Features	Number of cases	MGLL expression		P
		High	Low	
Age				
≥ 60	82	39	43	0.975
< 60	74	35	39	
Gender				
Male	93	46	47	0.538
Female	63	28	35	
Tobacco use				
No	84	43	41	0.310
Yes	72	31	41	
Histology grade				
G1/G2	71	27	44	0.037
G3	85	47	38	
TNM stage				
I+II	83	33	50	0.041
III	73	41	32	

### MGLL Knockdown Inhibits LUAD Cell Proliferation and Tumor Growth *in Vitro* and *in Vivo*

**Figure 2:**
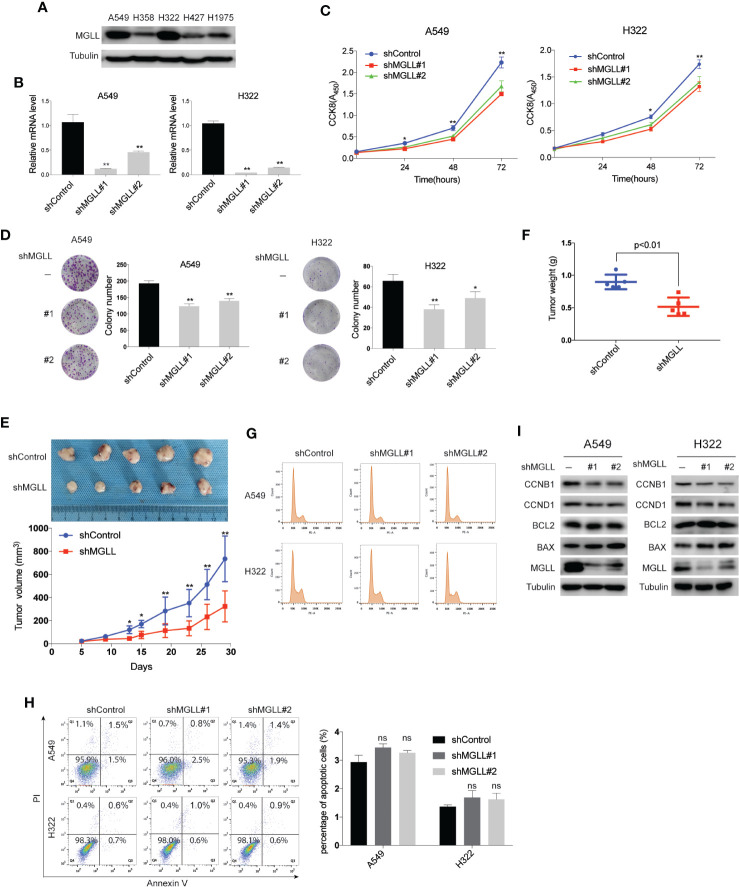
MGLL knockdown inhibits LUAD cell proliferation and tumor growth. **(A)** Western blot analysis to measure the levels of MGLL in 5 LUAD cell lines. **(B)** MGLL knockdown efficiency in LUAD cells was verified by qRT-PCR. **(C)** The proliferative capacity of MGLL-knockdown and control A549 and H322 cells was measured by the CCK8 assay. **(D)** Representative images from colony formation assays with shMGLL and control in A549 and H322 cells. Columns on the right show the mean number of clones from three independent experiments. **(E, F)** Images of excised xenograft tumors from nude mice subcutaneously injected with shMGLL or vector control A549 cells. Growth curves and average tumor weight for each group are shown. **(G)** Flow cytometric cell cycle analysis revealed that knockdown of MGLL decreases the percentage of S and G2/M phase cells. **(H)** Cell apoptosis analysis showed that the percentage of apoptotic cells was not significantly affected following MGLL knockdown in A549 and H322 cells (ns, not significant). **(I)** Western blot analysis to measure expression of the cell cycle proteins Cyclin D1 and Cyclin B1 and the apoptosis-related proteins BCL-2 and BAX in MGLL-knockdown and control cells. *P < 0.05, **P < 0.01.

To investigate the effect of MGLL on the malignant phenotypes of LUAD cells, we used shRNA to stably knockdown MGLL expression in the A549 and H322 cell lines, which display higher endogenous MGLL expression (**Figures 2A, B**). Cell viability was then evaluated by CCK-8 and colony formation assays. Our data show that MGLL depletion significantly attenuates both cell proliferation and colony formation in A549 and H322 cells, as compared to cells transfected with empty vector control (**Figures 2C, D**).

To further investigate the effects of MGLL knockdown on tumor growth *in vivo*, A549 cells with stable MGLL-knockdown or negative control cells were injected subcutaneously into the right flank of nude mice. Consistent with our *in vitro* data, we found that tumor size and weight were markedly reduced in the A549-shMGLL group compared to the control group (**Figures 2E, F**).

A previous study reported that knockdown of MGLL in colorectal cancer cells inhibits tumor cell proliferation and induces apoptosis *via* downregulation of Cyclin D1 and BCL-2 (18). Using flow cytometry, we tested whether a similar effect could be observed in LUAD cells. Indeed, we detected a reduced percentage of MGLL-knockdown cells in the S and G2/M phases, as compared to the negative control, for both A549 and H322 cells (**Figure 2G**). However, cell apoptosis revealed that the percentage of apoptotic cells was not significantly affected following MGLL knockdown (**Figure 2H**). We then measured the protein levels of cell proliferation and apoptosis-related proteins by western blot and found that MGLL knockdown attenuates the expression of both Cyclin D1 and Cyclin B1; whereas the expression levels of the apoptosis-related proteins BCL-2 and BAX are not significantly affected by MGLL knockdown (**Figure 2I**). Taken together, our results suggest that enhanced MGLL expression promotes lung cancer cell proliferation and tumor growth *in vitro* and *in vivo*.

### MGLL Knockdown Inhibits LUAD Cell Migration and Invasion *in Vitro* and *in Vivo*

**Figure 3:**
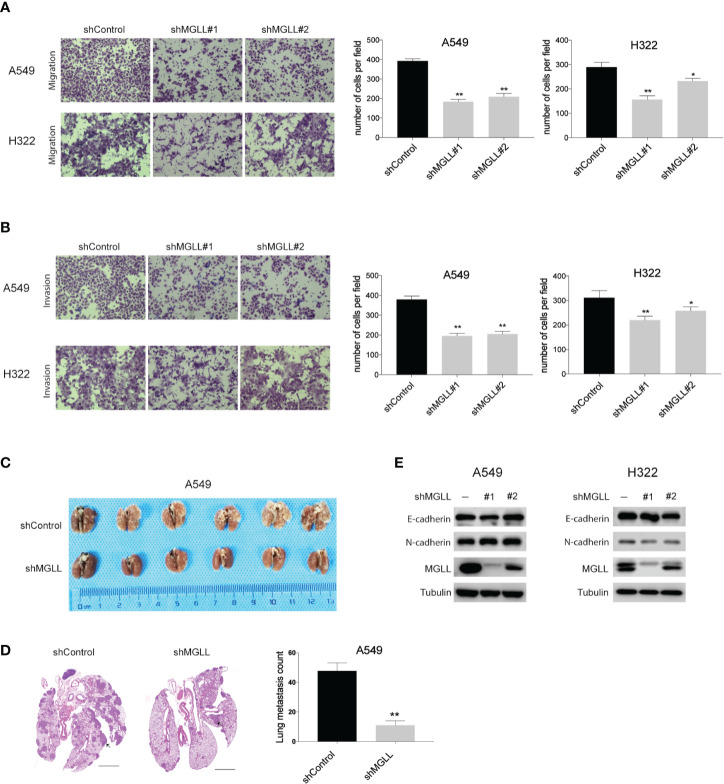
Knockdown of MGLL inhibits migration and invasion of LUAD cells. **(A, B)** Transwell assays to measure the migration and invasion activities of shMGLL and control A549 and H322 cells. **(C)** Representative images of lung tissues isolated from mice injected with shMGLL or vector control A549 cells *via* the tail vein. **(D)** Representative hematoxylin and eosin-stained images of lung tissues from shMGLL and control mice, and metastatic nodules are indicated by arrow heads; scale bar = 2 mm. **(E)** Western blot analysis to measure expression of EMT markers in MGLL-knockdown and control A549 and H322 cells. *P < 0.05, **P < 0.01.

To investigate the effect of MGLL on the migration and invasion of LUAD cells, we performed transwell assays with stable MGLL-knockdown and control cells. We found that both the migratory and invasive capabilities of A549 and H322 cells are significantly suppressed by MGLL knockdown relative to the control (**Figures 3A, B**).

We then injected A549 cells containing the shMGLL or control plasmid into the tail veins of NOD-SCID mice and quantified the number of metastatic nodules in lungs after 8 weeks. We found that mice injected with A549-shMGLL cells displayed fewer pulmonary metastatic nodules compared to those injected with the vector control cells (**Figure 3C**). Hematoxylin and eosin (H&E) stained images of lung tissue specimens are shown in **Figure 3D**. We also measured the expression levels of the epithelial-to-mesenchymal transition (EMT) markers E-cadherin and N-cadherin, which were reported to be regulated by MGLL in hepatocellular carcinoma (17). However, our results indicate that the expression levels of both proteins are not significantly affected by MGLL knockdown (**Figure 3E**).

### MGLL Is Involved in Multiple Cellular Pathways and Regulates the Expression of MMP14

**Figure 4:**
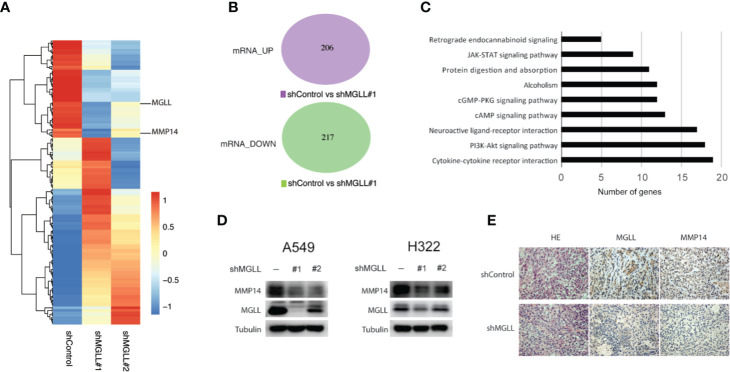
MGLL knockdown inhibits expression of MMP14 in LUAD cells. **(A, B)** Heatmap and Venn diagram showing the up- and downregulated genes following MGLL knockdown in A549 cells, as determined by RNA-seq analysis. **(C)** Top-ranked KEGG pathway terms associated with up- and downregulated genes. **(D)** Western blot analysis measuring the protein levels of MGLL and MMP14 in MGLL-knockdown and control A549 and H322 cells. **(E)** Representative IHC images of MGLL and MMP14 staining in MGLL-knockdown and control xenograft tumors; scale bar = 50 µm.

To better understand the molecular mechanisms underlying the role of MGLL in LUAD proliferation and metastasis, we performed RNA-seq analysis to uncover the transcriptional profiles of A549 cells following MGLL knockdown (**Figures 4A, B**, **Supplementary Data1**). Consistent with the metabolic role of MGLL, KEGG pathway analysis indicated that MGLL primarily affected biological processes such as cytokine-cytokine receptor interaction, PI3K-Akt signaling pathway, neuroactive ligand-receptor interaction, and retrograde endocannabinoid signaling (**Figure 4C**). We then carefully examined the list of upregulated and downregulated mRNAs and found that expression of MMP14 is significantly decreased following MGLL knockdown (**Figure 4A**). Proteins in the matrix metalloproteinase family are known to be involved in the breakdown of extracellular matrix components and often associated with tumor invasion. Consistent with our transcriptional profiling, western blot analysis revealed that expression of MMP14 is significantly decreased following MGLL knockdown in A549 and H322 cells (**Figure 4D**). Moreover, IHC staining of tissue from the A549 xenograft model confirmed this result (**Figure 4E**).

### MMP14 Is Upregulated and Associated With Poor Outcome in LUAD Tissues

**Figure 5:**
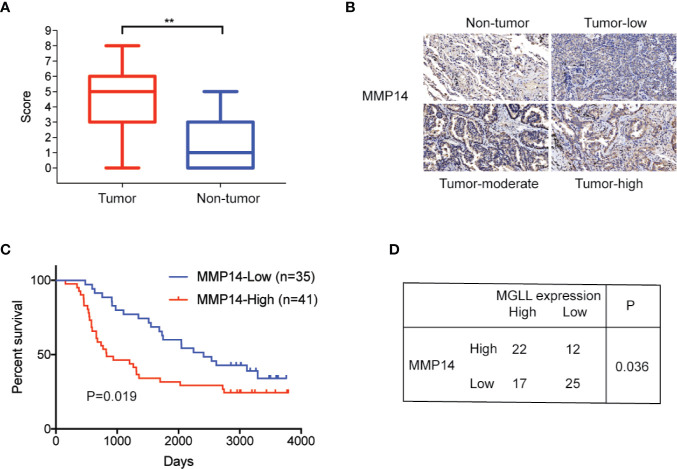
MMP14 is overexpressed in LUAD, and protein levels are correlated with MGLL expression. **(A)** IHC staining demonstrates that MMP14 is overexpressed in LUAD relative to adjacent non-tumor tissues (**P < 0.01). **(B)** Representative images from IHC staining of MMP14 in LUAD and non-tumor tissues; scale bar = 50 µm. **(C)** Kaplan-Meier analysis indicates that high MMP14 expression is predictive of poor overall survival. **(D)** Expression of MMP14 is correlated with MGLL expression, as demonstrated by the chi-square test (P = 0.036).

To further assess the clinical significance of MMP14 in lung cancer, we measured the expression levels of MMP14 in 76 LUAD and paired adjacent non-tumor tissues using IHC. Our results revealed that MMP14 is significantly overexpressed in tumor tissues compared to non-tumor tissues (**Figures 5A, B**), and high MMP14 expression is significantly correlated with poor overall survival (*P* = 0.019, **Figure 5C**). In addition, we detected a positive correlation between expression of MGLL and MMP14 in the 76 tumor tissue samples (P = 0.036, **Figure 5D**). Taken together, our data indicate that MGLL promotes cell proliferation and metastasis and plays an oncogenic role in LUAD.

## Discussion

The role of lipid metabolism in cancer has gained increasing attention in recent years. In particular, it has become well recognized that aberrant expression of genes involved in fatty acid synthesis or fatty acid oxidation correlate with malignant phenotypes, including metastasis and drug resistance (22–24). As described above, MGLL is an important enzyme in the process of lipolysis, and the oncogenic roles of MGLL have been validated in multiple tumors. In this study, we present both clinical and experimental evidence demonstrating that MGLL is an oncogenic factor that promotes LUAD tumor cell proliferation and metastasis.

MGLL upregulation has been reported in several tumor tissues, including hepatocellular carcinoma and colorectal cancer (17, 18). Here we showed that MGLL is upregulated in LUAD tissues *via* IHC staining, and MGLL expression levels were significantly correlated with overall survival. These results indicate that MGLL may serve as a valuable prognostic biomarker for LUAD.

MGLL can promote cancer cell proliferation in many ways, such as *via* fatty acid accumulation, regulation of the cell cycle, and inhibition of apoptosis (16, 18). We therefore evaluated the role of MGLL in cell proliferation in A549 and H322 cells and found that MGLL knockdown inhibits cancer cell proliferation both *in vitro* and *in vivo*. We further found that MGLL regulates expression of Cyclin B1 and Cyclin D1, which are required for cell cycle transition. Interestingly, the expression of apoptosis-related proteins BCL-2 and BAX was not significantly affected following MGLL knockdown, suggesting that MGLL may not affect apoptosis in LUAD cells.

Previous studies have reported that MGLL can regulate and promote cell metastasis through the involvement of multiple pathways. In hepatocellular carcinoma, for example, MGLL promotes disease progression *via* NF-kB-mediated EMT transition (17). The role of MGLL in colorectal cancer oncogenesis, however, is controversial. Ye L. et al. demonstrated that increased MGLL levels have a tumor-promoting effect in colorectal cancer cell lines (18). Conversely, Sun H. et al. detected reduced MGLL expression in colorectal cancer tissues and reported a tumor suppressive effect for MGLL overexpression in colon cancer cells (25). Here, our data indicate that MGLL can promote lung cancer cell metastasis, both *in vitro* and *in vivo*. However, we found that expression of EMT markers, including E-cadherin and N-cadherin, was not significantly affected following MGLL knockdown in A549 and H322 cells. To further elucidate the mechanisms underlying the oncogenic role of MGLL in LUAD, we performed RNA-seq and identified genes and pathways regulated by MGLL. Our results indicate that MGLL is primarily involved in processes, such as cytokine-cytokine receptor interaction, PI3K-Akt signaling pathway, neuroactive ligand-receptor interaction, and retrograde endocannabinoid signaling (**Figure 4C**). Previous studies have also reported that MGLL is involved in endocannabinoid signaling (26–29), and notably, the endocannabinoid system has been found to play a significant role in cancer development and may serve as a therapeutic target (30–33). Although an investigation into the role of the endocannabinoid system in LUAD was beyond the scope of this study, future research should be aimed at exploring a possible role for MGLL in modulating endocannabinoid signaling in LUAD.

Among the MGLL-regulated genes uncovered *via* RNA-seq, we found that MMP14 is significantly downregulated following MGLL knockdown. These data are consistent with reports showing that MMP14 is often upregulated during tumor progression, and its upregulation predicts the invasive potential of many cancers (34–36). We therefore measured the expression of MMP14 in LUAD tissues and found that this protein is upregulated in LUAD relative to non-tumor tissues, and like MGLL, its expression is significantly correlated with overall survival. More importantly, using the chi-square test, we further found that the expression of MMP14 is significantly correlated with MGLL expression.

However, in this study, we did not investigate the roles of MGLL in lung squamous cell carcinoma (LUSC), which is another broad subtype of non-small cell lung cancer. LUSC is different from LUAD in many ways, including gene expression profiles and genomic alterations (37). Thus, further study is needed to characterize the roles of MGLL in LUSC.

In summary, to our knowledge, we present the first study demonstrating that MGLL plays an oncogenic role in LUAD. Critically, we find that knockdown of MGLL inhibits the expression level of Cyclin B1, Cyclin D1 and MMP14. LUAD tissues with high MGLL and MMP14 expression are associated with poor overall survival. Our results therefore suggest that MGLL may serve as both a potential prognostic marker and a therapeutic target for LUAD patients.

## Data Availability Statement

The datasets presented in this study can be found in online repositories. The names of the repository/repositories and accession number(s) can be found below: BioProject, PRJNA606140 (https://www.ncbi.nlm.nih.gov/sra/?term=PRJNA606140).

## Ethics Statement

This study was approved by the Research Ethics Committee of the Cancer Hospital, Chinese Academy of Medical Sciences. Written informed consent to participate in this study was provided by the participants.

## Author Contribution

HZ: methodology, data analysis, writing original draft. WG, FZ, RL, YZ, and FS: methodology, patient information collection. XF, JW: investigation, data curation. FT, SG: investigation. YG and JH: conceptualization, supervision. All authors contributed to the article and approved the submitted version.

## Funding

This study was supported by the National Key R&D Program of China (2017YFC1308700,2017YFC1311000); CAMS Initiative for Innovative Medicine (2017-I2M-1-005, 2017-I2M-2-003, 2019-I2M-2-002); Non-profit Central Research Institute Fund of Chinese Academy of Medical Sciences (2018PT32033); Innovation team development project of Ministry of Education (IRT_17R10). R&D Program of Beijing Municipal Education commission (KJZD20191002302)

## Conflict of Interest

The authors declare that the research was conducted in the absence of any commercial or financial relationships that could be construed as a potential conflict of interest.
