# Supplementary material for: Monoacylglycerol Lipase Knockdown Inhibits Cell Proliferation and Metastasis in Lung Adenocarcinoma
Source: Front Oncol. 2020 Dec 9;10:559568. doi: 10.3389/fonc.2020.559568 (PMC7756122; doi:10.3389/fonc.2020.559568)
Supplement: Supplementary file 1 [file Table_1.docx]

|  | Univariate analysis HR(95% CI) | P | Multivariate analysis  HR(95% CI) | P |
| --- | --- | --- | --- | --- |
| Age | 1.914(1.300-2.819) | 0.001 | 1.929(1.304-2.846) | 0.001 |
| (≥60 vs <60) |  |  |  |  |
| Gender | 0.777(0.527-1.146) | 0.203 |  |  |
| (Male vs Female) |  |  |  |  |
| Tobacco use | 1.098(0.753-1.599) | 0.628 |  |  |
| (Yes vs No) |  |  |  |  |
| Histology grade | 1.681(1.141-2.476) | 0.008 | 1.299(0.863-1.954) | 0.210 |
| (3 vs 1/2) |  |  |  |  |
| TNM stage | 1.959(1.339-2.866) | 0.000 | 1.756(1.177-2.622) | 0.006 |
| (III vs I/II) |  |  |  |  |
| MGLL | 1.475(1.012-2.151) | 0.042 | 1.379(0.940-2.024) | 0.100 |
| (High vs Low) |  |  |  |  |

Supplementary table 1. Univariate and multivariate analysis of overall survival among patients with LUAD.
